# Predicting Self-Initiated Marijuana Use Cessation among Youth at Continuation High Schools

**DOI:** 10.3389/fpsyt.2013.00069

**Published:** 2013-07-26

**Authors:** Melissa A. Little, Donna Spruijt-Metz, Pallav Pokhrel, Ping Sun, Louise Ann Rohrbach, Steve Sussman

**Affiliations:** ^1^University of Hawai’i Cancer Center, Honolulu, HI, USA; ^2^Keck School of Medicine, Institute for Prevention Research, University of Southern California, Los Angeles, CA, USA

**Keywords:** marijuana, cessation, adolescents, youth, cannabis, self-initiated, predictors

## Abstract

The current article reports a large scale study of the prediction of marijuana use cessation among individuals attending alternative high schools who were regular users at baseline. Based on the Triadic Influence Theory, predictors of marijuana use cessation at 1-year follow-up were organized by type of influence (e.g., interpersonal, cultural and attitudinal, and intrapersonal) and level of influence (e.g., distal and ultimate). Among the 522 students who were past 30-day marijuana users at baseline, quitting was defined as having not used marijuana in the last 30 days at 1-year follow-up (43% of baseline users). To account for the level of influence we employed a theory-based analytic strategy, hierarchical regression. In the final multivariate model, lower level of baseline marijuana use and less of a likelihood to endorse pro-drug-use myths remained predictors of marijuana use cessation 1-year later. Implications of these findings include the need to develop cessation programs that reduce psychological dependence on marijuana use, and correct cognitive misperceptions about drug use in order to help adolescents make decisions that lead to health-promoting behaviors.

## Introduction

Marijuana is the most commonly used illicit substance among youth in the United States (1). Since 2009, the 30-day prevalence of marijuana use among youth has steadily risen to 31%, while perceived risk and disapproval of marijuana have declined (1). Recent data show that nearly one in 15 high schools seniors is a daily or near-daily marijuana user (1). Given the recent legislation that legalizes the recreational use and sale of marijuana in Colorado and Washington, one could speculate that these trends in marijuana use among American youth will continue to rise. Unfortunately, most adolescents that desire to quit are unsuccessful (2).

In order to develop effect marijuana use cessation programs, it is essential that we improve our understanding of factors related to self-initiated marijuana use cessation. However, few studies have been conducted examining predictors of marijuana use cessation among teens (3–7) and young adults (8–12).The purpose of the current study was to apply a theoretical framework to the prediction of marijuana use cessation at 1-year follow-up among a sample of adolescents attending alternative high schools who were regular users at baseline.

The Theory of Triadic Influence (13) organizes predictors of adolescent substance use into three distinct types of influence: (1) intrapersonal, (2) cultural/attitudinal, and (3) social/interpersonal. Within each type of influence, predictors are further ordered into level of influence (e.g., the adolescent’s ability to control the influence and its effect on the behavior). Levels of influence include, (1) proximal, most control, and most direct influence on behavior, (2) distal, and (3) ultimate, least control, and most indirect influence on behavior. In previous studies, this theory has provided a framework for explaining variance in substance use among samples of adolescents (14–17).

Intrapersonal correlates of drug use are those that describe personality traits, affective states, and beliefs about one’s ability to either use or avoid substances (13). Depressed adolescents and adolescents scoring lower on a measure of delinquency have been found to be more likely to quit use of marijuana (11). Cultural and attitudinal correlates of drug use include beliefs and evaluations regarding substance use, as well as general values and behaviors that contribute to substance use (13). Endorsing negative social and psychological consequences of marijuana use (3, 6), having alternative interests (3, 6), holding unfavorable attitudes about the acceptability of drug use (5, 6), and rating one’s health as excellent (9) have all been associated with marijuana use cessation among youth and young adults. Social and interpersonal variables are those that operate within the subject’s social environment, generally as reported by the subject, and influence one’s perceptions of one’s social world. Participation in adult social roles (e.g., marriage, being a parent) (7, 9–12), less peer use and approval (5–9), less victimization (5), and using marijuana for social reasons (4, 9, 11) have also been associated with marijuana use cessation among youth and young adults.

The best predictor of future marijuana use is past behavior. A number of studies have found that light marijuana smoking at baseline was associated with cessation at follow-up (4, 7, 9, 11). Since heavier marijuana users suffer from greater withdrawal symptoms (e.g., cravings, irritability, sleep difficulty, decreased appetite) (18), this discomfort could deter them from making quit attempts and experiencing cessation success (2). Other factors associated with marijuana use cessation among adolescents and young adults include older age at initiation and cessation (3–5), the lack of use of other illicit drugs (8), female gender (7, 9), higher income (9), and steady employment (10).

The purpose of the current study was twofold. First, we sought to examine the relationships between baseline demographic, intrapersonal, cultural/attitudinal, social/interpersonal, and drug-use variables, and marijuana use cessation 1-year later. We did not have any predictors that fell within the proximal level of influence; however, we did explore factors within the distal and ultimate levels of influence. Secondly, because we hypothesized that heavier smokers would experience greater discomfort during the cessation attempt, we wanted to predict cessation controlling for baseline level of use which is likely to be the best predictor of cessation. Therefore, we explored how adjusting for baseline level of marijuana use affected the relationships between intrapersonal, cultural/attitudinal, and social/interpersonal variables and marijuana use cessation. We hypothesized that intrapersonal, cultural/attitudinal, and social/interpersonal variables would be found to be associated with marijuana use cessation, however the strength of the associations would diminish once the statistical models accounted for baseline level of marijuana use.

## Materials and Methods

### Subjects

The sample consisted of 522 past 30-day marijuana users participating in a field trial conducted to test the efficacy of a substance abuse prevention program [see (19, 20)]. A total of 24 continuation high schools in four counties in southern California were recruited as a convenience sample. Continuation high schools, otherwise known as alternative high schools, are schools designated for youth who have transferred out of the regular school system due to functional problems (e.g., behavior problems, drug use, lack of credits). Schools were randomly assigned to one of the following conditions: (1) control condition; (2) TND program condition (TND Only); or (3) TND + motivational interviewing (TND + MI) condition. Prior to enrollment, parental informed consent and subject assent were required for youth under age 18, and informed consent was obtained from participants over the age of 18. Within each school, at least two classrooms were selected to participate in the study. Of the 2,397 students enrolled in the selected classes, 1,694 (70.7%) were consented to participate in the study and 1,676 students completed the baseline survey. Reasons for subject-level decline were parent decline of consent (0.8%), student decline of consent or assent (5.1%), and parental non-response (23.4%). Of the 1676 students who completed the baseline surveys, 1186 (70.8%) completed 1-year follow-up surveys. Of the 778 past 30-day marijuana users at baseline, 522 (67%) completed 1-year follow-up surveys. Students who completed both pre- and 1-year follow-up surveys and reported they had used marijuana in the past 30 days at baseline constitute the present sample for analysis.

Students completed close-ended, self-report questionnaires during regular classroom sessions at baseline and approximately 1-year after the immediate posttest in class. Absent students were left an absentee packet with instructions for completing the survey. Surveys included measures of demographic characteristics, behavioral items, and psychosocial correlates of substance use, and took approximately 20–30 min to complete. All study procedures, including informed consent, were approved by the University of Southern California’s institutional review board.

At baseline, subjects ranged in age from 14 to 20 years (mean age of 16.7, SD = 0.91). Sixty percent were male. The ethnic/racial distribution of the sample was as follows: 11.6% White, 62.6% Latino, 5.3% African American, 16.0% Mixed Ethnicity, and 4.5% Other Ethnicity (including Asian, Native American, and “other”). At baseline, 63.6% reported use of cigarettes, 81.7% reported use of alcohol, and 47.9% reported use of hard drugs in the past 30 days. At baseline, subjects reported smoking marijuana on average 14.1 days (SD = 12.2) in the past 30 days. At 1-year follow-up, 43.5% of the sample reported no marijuana use in the past 30 days.

### Measures

#### Demographics

Demographic items included age (in years), gender, and ethnicity (a four-level categorical variable with response categories being White/Caucasian, Latino/Hispanic, African American/Black, or Mixed Ethnicity). This categorical variable was coded with three dummy indicators: White, Latino, and African American.

#### Marijuana use behavior

The main outcome in the study was past 30-day marijuana use cessation between pretest and 1-year follow-up. At both time points, subjects were asked “How many times in the last month have you used marijuana?” Responses were reported on 12-point scales, starting at “0 times,” increasing in intervals of 10 (e.g., “1–10 times,” “11–20 times”) with the last (12th) category being “over 100 times.” The “quit” status was defined as reporting “0” times of use in the last 30 days.

#### Other drug-use measures

Substance use items included 30-day use of cigarettes, alcohol, and “hard drugs.” A hard drug use score was created, consisting of the sum of 30-day use across cocaine, hallucinogens, stimulants, inhalants, ecstasy, tranquilizers, and “other” drugs (an item that included “PCP, steroids, GHB, K, etc.”; alpha = 0.89). The drug-use questionnaire items are the type used in the Monitoring the Future studies (1, 21) and previous work showing evidence of adequate test-retest reliability and/or internal consistency (22–24). The wording and response options of the other drug-use items were the same as that of the marijuana use item.

#### Triadic influence theory-related measures

##### Interpersonal influences

*Family conflict* was assessed through five items, on 4-point scales from “Describes my family ‘very well’ to ‘not at all”’ such as “We fight a lot in our family” [α = 0.64; e.g., (25, 26)]. Four single item measures of five closest *friends*’ *use of cigarettes*, *alcohol*, *marijuana*, *and hard drugs* each had six response options ranging from 0 to 5 friends [e.g., (15)]. *Family member drug abuser* was measured through one dichotomous item, “Do any members of your family abuse drugs or alcohol?” (0 = “no” or 1 = “yes”). Additionally, two items included *whom the student lives with* (both parents, only mother, only father, sometimes mother and sometimes father, other, or alone; coded as living with both parents or not), and *having one or more children* (0 = “no” or 1 = “yes”).

##### Cultural and attitudinal influences

*Socioeconomic status* was assessed by rooms-per-person in the home, calculated as the quotient of total number of rooms (except kitchen, bathrooms, closets, or laundry rooms) divided by the number of people living in the home. *Acculturation* was assessed through four items measuring language preference on 5-point scales from “only English” to “only another language (not English)” [α = 0.86; (27, 28)]. *Morality of drug use* was assessed through four items on 4-point scales such as “How wrong is it to use drugs?” from “it is not wrong at all” to “it is very wrong” [α = 0.90; e.g., (26)]. *Pro-drug-use myths* (29), were measured through four items each with a two-option forced-choice response. A sample item is “What happens when a person gets used to a drug?” [(a) one has learned how to enjoy using the drug, to control its effect, OR (b) body warning signals are giving up and addiction is beginning] (alpha = 0.55). The importance of *health as a value* was assessed through three items on 4-point scales from “not at all” to “very much” such as “How important is it for people to be physically healthy?” [α = 0.79; e.g., (30)].

Finally, two constructs of *emerging adulthood* from the Inventory of the Dimensions of Emerging Adulthood (IDEA) scale were assessed: experimentation/possibilities and feeling in-between (31). Respondents were asked to “Please think about this time in your life. When we say ‘this time,’ we mean what is going on right now, plus what has gone on in the last few years, plus what you think your life will be like in the next few years. Think about a 5-year period of time, with right now in the middle. For each question below, mark the box that best describes this time in your life. Be sure to put only one check mark per line.” Responses were 4-point scales from “definitely not” to “definitely yes.” A set of five items measured *experimentation/possibilities*, such as “Time of exploration?” (α = 0.79). Three items assessed *feeling in-between*, such as “Time of feeling adult in some ways but not in others?” (α = 0.68).

##### Intrapersonal influences

*Social self-control* was measured using eight items (32), which were on 4-point scales from “never” to “always” such as “I enjoy arguing with people,” “If I think something one says is stupid I tell them so,” and “My mouth gets me in trouble a lot” (alpha = 0.73). *Depressive symptoms* were measured using five items from the short CES-D scale, measured on 4-point scales from “less than 1 day” to “5–7 days” in last week, such as “How often did you feel depressed in the last 7 days?” [α = 0.73; see (33)]. Four items measured *assertiveness* (26, 34) on 4-point scales from “never” to “always,” such as “It is hard for me to express an opinion that differs from what the person I am talking to is saying” (alpha = 0.60). *Coping* was measured through four constructs, each with three item, 5-point scales from “never” to “always” [e.g., (35)]. *Anger coping* included items such as “I yell and scream at someone” (α = 0.77). *Social support coping* included items such as “I get emotional support from my mother/father (α = 0.87). *Cognitive coping* included items such as “I think about the choices before I do anything” (α = 0.85). *Avoidance coping* included items such as “I daydream about better times” (α = 0.79). *Decision-making* was measured with two constructs each with three items on 4-point scales from “always” to “never” (36). *Decision-making confidence* included items such as “I like to make decisions myself” (α = 0.70). *Decision-making avoidance* included items such as “I prefer to leave decisions to others” (α = 0.75).

### Data analysis

#### Assessment of attrition bias

To determine the potential attrition bias, a comparison was made between the current analytic sample (*N* = 522) to the baseline past 30-day marijuana users lost to follow-up (*N* = 256) on baseline measures of demographics and use of substances other than marijuana. The comparisons utilized chi-square or *t*-test models to indicate statistically significant differences (*p* value at the 0.05 level, two-tailed). Relative to the study dropouts, the retained sample was more likely to be slightly younger (16.6 versus 16.8) and smoke cigarettes on fewer days in the past 30 days (7.2 versus 9.4). There were no significant differences on any of the other characteristics.

#### Prediction of marijuana use cessation

We employed hierarchical logistic regression analyses (37) to examine the associations between the predictors as assessed at baseline and marijuana use cessation at 1-year follow-up. The dichotomous quit status outcome was defined as “yes” if the subject reported not using marijuana in the past 30 days. The dichotomous outcome analysis was completed using generalized mixed-logistic modeling (38). School was treated as a random effect, which statistically accounts for the intra-class correlation within clustered units (school) on computed significance levels.

In the first step of our analyses, we established eight sets of predictors based on type and level of influence, including (1) five demographic variables, (2) four baseline drug-use variables, (3) two ultimate interpersonal variables, (4) three distal interpersonal variables, (5) two ultimate cultural/attitudinal variables, (6) five distal cultural/attitudinal variables, (7) one ultimate intrapersonal variable, and (8) eight distal intrapersonal variables. In the second step, we ran two generalized mixed-logistic regression models that included the first two predictor sets demographic characteristics and baseline drug use, to predict marijuana use cessation at 1-year follow-up. Because we assumed that distal factors were more strongly related to marijuana cessation than ultimate factors, in the third step, we employed hierarchical mixed-logistic regression analyses for the interpersonal, cultural/attitudinal, and intrapersonal predictor set models. Utilizing hierarchical regression allowed us to avoid improperly adjusting for distal factors in the relationship between ultimate factors and marijuana cessation, and consequently diminishing the effects of ultimate factors. Therefore, for each type of influence, first we ran models for predictors at the ultimate level of influence. Then, we ran models for predictors at the distal level of influence, controlling for ultimate predictors.

Lastly, we ran two final generalized mixed-logistic regression model in which we entered all of the predictors across the predictor sets that were significant at the level of *p* < 0.05. However, because past marijuana use is the best predictor of future use, we wanted to predict cessation controlling for baseline level of use. Therefore, in the first final model, level of baseline marijuana use was excluded, and in the second final model, level of baseline marijuana use was included. All regression models controlled for experimental condition (nuisance variable in the present study). Variables were standardized (mean = 0 and standard deviation = 1). Odds ratios and 95% confidence intervals were reported using two-tailed significance tests. To compare the goodness-of-fit of the final logistic regression models we calculated the Hosmer–Lemeshow Goodness-of-Fit test, the relative operating characteristic (ROC) curve analysis, and compared the log likelihoods for the full versus the null models. Analyses were conducted using the SAS (v.9.1.3) statistical package (39).

## Results

Results of the regression models with baseline demographics and drug-use predicting marijuana use cessation are shown in Table 1. Males were less likely than females to quit marijuana use at 1-year follow-up (*p* < 0.05). Also, being of Latino ethnicity and having a lower level of baseline marijuana use predicted marijuana use cessation at 1-year follow-up (*p* < 0.05).

**Table 1:** **Baseline demographics and drug use predicting marijuana cessation at the 1-year follow-up**.

Predictors	OR (95% CI)
**DEMOGRAPHICS MODEL**	
Age	0.98 (0.81, 1.19)
Male	0.74 (0.62, 0.89)*
White ethnicity	1.05 (0.83, 1.32)
Latino ethnicity	1.40 (1.10, 1.78)*
African American ethnicity	1.10 (0.88, 1.37)
**BASELINE DRUG USE MODEL**	
30-day cigarette use	0.83 (0.65, 1.06)
30-day alcohol use	1.09 (0.86, 1.37)
30-day marijuana use	0.50 (0.40, 0.63)*
30-day hard drug use	1.09 (0.86, 1.38)

*All values are standardized odds ratios and 95% confidence intervals*.

*p < 0.05.

Table 2 shows the results from the hierarchical regression models that examined sets of predictors of marijuana use cessation that were established based on the Triadic Influence Theory (TTI). Among the ultimate interpersonal variables, having at least one child was predictive of marijuana use cessation (*p* < 0.05). Among the distal interpersonal variables, friends’ substance use was inversely related to marijuana use cessation (*p* < 0.05). Among the ultimate cultural/attitudinal variables, being less acculturated was predictive of marijuana use cessation (*p* < 0.05). Among the distal cultural/attitudinal variables, having a lower likelihood to endorse pro-drug-use myths and having weaker beliefs that this was a period of life for experimentation were predictors of marijuana use cessation 1-year later (*p*’s < 0.05). The ultimate intrapersonal variable, social self-control, was not predictive of marijuana use cessation. Among the distal intrapersonal variables, having both avoidant and confident decision-making styles were predictive of marijuana use cessation (*p*’s < 0.05).

**Table 2:** **Summary of hierarchical regression analysis for social/interpersonal variables predicting marijuana cessation at the 1-year follow-up**.

Level of Influence	Types of influence					
	Interpersonal	OR (95% CI)	Cultural/attitudinal	OR (95% CI)	Intrapersonal	OR (95% CI)
Ultimate	Living with both parents	1.11 (0.93, 1.33)	Socioeconomic status	0.83 (0.68, 1.01)	Social self-control	1.06 (0.87, 1.29)
	Have ≥ 1 children	1.25 (1.01, 1.54)*	Acculturation	1.41 (1.16, 1.71)*		
Distal	Living with both parents	1.08 (0.88, 1.32)	Socioeconomic status	0.90 (0.74, 1.09)	Social self-control	1.03 (0.81, 1.31)
	Have ≥ 1 children	1.17 (0.94, 1.46)	Acculturation	1.40 (1.13, 1.73)*	Depressive symptoms	0.99 (0.79, 1.23)
	Friends’ substance use	0.72 (0.58, 0.90)*	Morality of drug use^1^	1.21 (0.97, 1.52)	Assertiveness	0.92 (0.73, 1.15)
	Family conflict	0.83 (0.67, 1.04)	Pro-drug-use myths^2^	0.72 (0.58, 0.89)*	Anger coping	0.98 (0.76, 1.27)
	Family member drug abuser	0.94 (0.76, 1.16)	Health as a value^3^	1.05 (0.83, 1.33)	Cognitive coping	1.03 (0.82, 1.29)
			Emerging adulthood		Avoidance coping	1.14 (0.91, 1.43)
			Experimentation/possibilities	0.69 (0.53, 0.89)*	Social support coping Decision-making avoidance	1.02 (0.83, 1.25)1.27 (1.01, 1.59)*
			Feeling in-between	1.00 (0.79, 1.27)	Decision-making-confidence	1.36 (1.08, 1.70)*

All values are standardized odds ratios and 95% confidence intervals. *p < 0.05.

*^1^Scale is drug use is not wrong (low) to drug use is wrong (high)*.

*^2^Higher value denotes pro-drug-use endorsement*.

*^3^Scale is disagree (low) to agree (high) regarding health as a value*.

The final multivariate regression model that did not include level of baseline marijuana use revealed that having fewer friends who use substances, endorsing fewer pro-drug-use myths, and having weaker beliefs that this was a period of life for experimentation were significant predictors of marijuana use cessation at 1-year follow-up (*p*’s < 0.05) (see Table 3). In the final multivariate regression model that included level of baseline marijuana use, having lower levels of baseline marijuana use and a lower likelihood to endorse pro-drug-use myths remained predictors of marijuana use cessation 1-year later (*p*’s < 0.05).

**Table 3:** **Final multivariate models: interpersonal, cultural/attitudinal, and intrapersonal variables predicting marijuana cessation (significant at <0.10)**.

Predictors	Excluding baseline marijuana use	Including baseline marijuana use
30-day marijuana use	–	0.68 (0.52, 0.85)*
Male	0.83 (0.68, 1.02)	0.89 (0.73, 1.13)
Latino	1.12 (0.88, 1.42)	1.16 (0.90, 1.49)
Have one or more children	1.17 (0.94, 1.46)	1.26 (0.98, 1.61)
Friends’ substance use	0.79 (0.64, 0.98)*	0.87 (0.69, 1.10)
Acculturation	1.22 (0.96, 1.55)	1.08 (0.84, 1.39)
Pro-drug-use myths^2^	0.73 (0.59, 0.90)*	0.77 (0.62, 0.95)*
Emerging adulthood
Experimentation/possibilities	0.77 (0.61, 0.98)*	0.82 (0.64, 1.05)
Decision-making avoidance	1.19 (0.95, 1.49)	1.18 (0.93, 1.49)
Decision-making self-confidence	1.19 (0.95, 1.50)	1.15 (0.90, 1.46)

*All values are standardized odds ratios and 95% confidence intervals*.

*p < 0.05

*^2^Higher value denotes pro-drug-use endorsement*.

To test the overall fit of the logistic regression models, we examined the likelihood ratio test which compares the log likelihoods for the full model versus the null model. For the model that did not include baseline marijuana use, the likelihood ratio test χ^2^ was 49.5, *df* = 9, *p* < 0.0001. For the model that included baseline marijuana use, the likelihood ratio test χ^2^ was 63.4, *df* = 10, *p* < 0.0001. Thus, the models showed a good fit to the data. We also calculated the Hosmer–Lemeshow Goodness-of-Fit test to assess whether the observed event rates matched the expected event rates in the model population (40). For the model that excluded baseline marijuana use, the Hosmer–Lemeshow Goodness-of-Fit test χ^2^ was 2.63, *df* = 8, *p* = 0.96. For the model that included baseline marijuana use, the Hosmer–Lemeshow Goodness-of-Fit test χ^2^ was 7.83, *df* = 8, *p* = 0.45. Both models showed a good fit to the data. In order to further evaluate the models we performed the ROC curve analysis. The ROC curve analysis assesses the power of a model’s predicted outcomes to discriminate between positive and negative cases (e.g., cessation versus use) in terms of the area under the ROC curve (i.e., plot of sensitivity versus 1-specificity), which is commonly referred to as the concordance index (*c*-statistic). We found that both of our models showed better-than-chance discriminating power in that the *c*-statistics for the models that excluded and included baseline marijuana use were 0.69 and 0.71, respectively (41).

## Discussion

In the present study we sought to fill a gap in the substance abuse literature by applying a theoretical framework, TTI (13), to assess the influence of demographic, drug use, intrapersonal, cultural/attitudinal, and social/interpersonal predictors of marijuana use cessation among a sample of adolescents. Using a theory-driven analytic approach, hierarchical regression analysis (37), we built upon previous research by assessing each of these types of influence at the appropriate level of influence (e.g., ultimate and distal).

Among the past 30-day marijuana users at baseline, 43% reported quitting marijuana use at 1-year follow-up. In the final multivariate model, several psychosocial predictors were negative predictors of marijuana use cessation at 1-year follow-up, including friends’ substance use, pro-drug-use myths, and beliefs that this was a period of life for experimentation. These findings are consistent with previous research (3, 5–9). Interestingly, after controlling for baseline marijuana use in the multivariate model, only baseline marijuana use and fewer pro-drug-use-myths were associated with marijuana use cessation. Given the strength of the association between baseline marijuana use and cessation, it is not surprising that after accounting for baseline use, many of the effects we saw in the first multivariate model disappeared. Based on previous research (2, 18), we hypothesized that heavier smokers would experience greater discomfort during the cessation attempt, and it is possible that these negative withdrawal symptoms (e.g., cravings, irritability, sleep difficulty) could have led many of the heavier baseline smokers to relapse. However, we did not assess relapse in the current study.

Another explanation for the elimination of effects after controlling for baseline marijuana use might be that these constructs could be statistically redundant when modeled simultaneously. Friends’ substance use and believing that this was a period of life for experimentation were significantly correlated with baseline marijuana use (*p*’s < 0.05). Therefore, one could speculate that the association between friends’ substance use and marijuana use cessation is mediated by baseline marijuana use. Adolescents who have friends that use marijuana may be more likely to use marijuana frequently and consequently, less likely to quit. However, mediation analyses were beyond the scope of this article. Future studies should examine whether these assumptions are true by employing mediation analysis.

Consistent with previous research (5, 7), we found a negative association between pro-drug-use myths and marijuana cessation in both final multivariate models. Drug-use myths encompass inaccurate expectancies or beliefs about drug characteristics and confusing drug effects with drug experiences (42). Cognitive restructuring of faulty or self-defeating cognitive structures has been shown to prevent drug use among high-risk youth (43, 44). Previous studies have found that Motivational Enhancement Therapy, Educational Feedback Control, and Cognitive Behavioral Treatment were effective in reducing marijuana use among adolescents (45, 46). Our findings suggest that these interventions could be strengthened by adding cognitive restructuring components.

The current study contributed to the substance abuse literature in several ways. The results of our paper are consistent with the few that have been done with teens on self-initiated marijuana cessation among a large sample of at-risk teens (*n* = 522 baseline self-reported marijuana users), adding to the literature on this topic. As there are not many such papers, and only one other with alternative (continuation) high school youth, this paper represents a welcome addition. Additionally, our paper uses hierarchical regression to assess components of TTI. This may be the first truly appropriate way to examine TTI, though only some support for the theory was provided. Further, the finding that likelihood to endorse pro-drug-use myths is a significant predictor of self-initiated cessation is a fairly novel finding and has implications for prevention efforts.

A limitation of the current study is we did not have any predictors that fell within the proximal level of influence. In addition, marijuana use cessation was not defined by self-reported quit status, but by inferred non-use status. This has been used as a proxy for self-reported quitting in previous studies (6, 8–11). While this methodology does not account for adolescents who may have made unsuccessful quit attempts, nor does it assume that quitting was an intentional act, because we observed significant associations between predictors of marijuana use cessation and quit status, we are confident that self-initiated quitting among adolescents was modeled. Another limitation to the current study is that we relied upon self-reported marijuana use without the use of biochemical validation (e.g., urine drug screens) of marijuana use at baseline or follow-up. Given the large sample size of the original study, it would have been impractical to obtain biological samples from all participants. However, we obtained Certificates of Confidentiality from the National Institutes of Health to protect our research information from forced disclosure, which was conveyed to study participants. Furthermore, the current study focuses on self-initiated marijuana cessation; therefore there would be little incentive for participants to lie. Our previous work in which we examined both anonymous and confidential data suggest that confidential self-reports are accurate among high school youth [e.g., (47)]. Others have found similar results (48, 49). It is true that in cigarette smoking cessation work with alternative high school youth, use of biochemical validation will lead to 2% lower reported quit rates (50); however, that is a small impact. In addition, use of biochemical validation with teens in research studies, in which biochemical validation is obtained voluntarily only, is bound to be biased because only cooperative youth will provide readings. Further, only written parental consent is permitted nowadays when biochemical validation of any drug use is being measured (other than possibly in anonymous, cross-sectional work). This would lead to inclusion of non-representative, small samples of teens in longitudinal survey work. Thus, most large survey research, including the Youth Risk Behavior Survey [e.g., (51)], do not include biochemical validation. Lastly, about a third of the baseline marijuana users were lost to follow-up. However, because those lost to follow-up were only significantly different from the retained sample on two factors (age and daily cigarette use), this limitation should not bias our results.

Efforts to develop adolescent marijuana use cessation programing should build on the results in the current study. Our findings support a motivation-skills-decision-making approach to adolescent marijuana use cessation (42). It is clear that adolescents with lower levels of baseline marijuana use have an easier time quitting. Additionally, cessation programing should include lessons that address correcting cognitive misperceptions about drug use.

## Conflict of Interest Statement

Dr. Steven Sussman receives royalties as miscellaneous income from USC for sales of Project TND, the project that provided the dataset on which the analysis was done. However, we do not believe that it would at all bias his judgment about the efficacy of TND or any other program, and is of negligible relevance to the present paper content.

## References

[1] Johnston LD O’Malley PM Bachman JG Schulenberg JE Monitoring the Future National Results on Adolescent Drug Use: Overview of Key Findings, 2011. Ann Arbor: Institute for Social Research, The University of Michigan (2012).

[2] Weiner MD Sussman S Mcculler WJ Lichtman KL Factors in marijuana cessation among high-risk youth. J Drug Educ (1999) 29:337–57 10.2190/PN5U-N5XB-F0VB-R2V1 10786412

[3] Goodstadt MS Sheppard MA Chan GC Non-use and cessation of cannabis use: neglected foci of drug education. Addict Behav (1984) 9:21–31 10.1016/0306-4603(84)90004-2 6611025

[4] Goodstadt MS Chan GC Sheppard MA Cleve JC Factors associated with cannabis nonuse and cessation of use: between and within survey replications of findings. Addict Behav (1986) 11:275–86 10.1016/0306-4603(86)90055-9 3739814

[5] Sussman S Dent CW Brief report one-year prospective prediction of marijuana use cessation among youth at continuation high schools. Addict Behav (1999) 24:411–7 10.1016/S0306-4603(98)00048-3 10400279

[6] Hansen WB McNeal RB Jr. Self-initiated cessation from substance use: a longitudinal study of the relationship between postulated mediators and quitting. J Drug Issues (2001) 31:957–76 10.1177/002204260103100408

[7] Sussman S Dent CW Five-year prospective prediction of marijuana use cessation of youth at continuation high schools. Addict Behav (2004) 29:1237–43 10.1016/j.addbeh.2004.03.024 15236829

[8] Kandel DB Marijuana users in young adulthood. Arch Gen Psychiatry (1984) 41:200–9 10.1001/archpsyc.1984.01790130096013 6607718

[9] Kandel DB Raveis VH Cessation of illicit drug use in young adulthood. Arch Gen Psychiatry (1989) 46:109–16 10.1001/archpsyc.1989.01810020011003 2913970

[10] Hammer T Vaglum P Initiation, continuation or discontinuation of cannabis use in the general population. Br J Addict (1990) 85:899–909 10.1111/j.1360-0443.1990.tb03720.x 2397317

[11] Chen K Kandel DB Predictors of cessation of marijuana use: an event history analysis. Drug Alcohol Depend (1998) 50:109–21 10.1016/S0376-8716(98)00021-0 9649962

[12] Leonard KE Homish GG Changes in marijuana use over the transition into marriage. J Drug Issues (2005) 35:409–29 10.1177/002204260503500209 17186062 PMC1715885

[13] Petraitis J Flay BR Miller TQ Reviewing theories of adolescent substance use: organizing pieces in the puzzle. Psychol Bull (1995) 117:67–86 10.1037/0033-2909.117.1.67 7870864

[14] Sussman S Dent CW Leu L The one-year prospective prediction of substance abuse and dependence among high-risk adolescents. J Subst Abuse (2000) 12:373–86 10.1016/S0899-3289(01)00053-0 11452840

[15] Rohrbach LA Sussman S Dent CW Tobacco, alcohol, and other drug use among high-risk young people: a five-year longitudinal study from adolescence to emerging adulthood. J Drug Issues (2005) 35:333–55 10.1177/002204260503500206

[16] Gunning M Sussman S Rohrbach LA Kniazev V Masagutov R Concurrent predictors of cigarette and alcohol use among U.S. and Russian adolescents. J Drug Educ (2009) 39:385–400 10.2190/DE.39.4.c 20443454

[17] Sussman S Rohrbach LA Spruijt-Metz D Barnett E Lisha N Sun P One-year prediction of pain killer use among at-risk older teens and emerging adults. J Drug Educ (2012) 42:195–210 10.2190/DE.42.2.e 23185838

[18] Budney AJ Hughes JR Moore BA Novy PL Marijuana abstinence effects in marijuana smokers maintained in their home environment. Arch Gen Psychiatry (2001) 58:917–24 10.1001/archpsyc.58.10.917 11576029

[19] Lisha NE Sun P Rohrbach LA Spruijt-Metz D Unger JB Sussman S An evaluation of immediate outcomes and fidelity of a drug abuse prevention program in continuation high schools: project towards no drug abuse (TND). J Drug Educ (2012) 42:33–57 10.2190/DE.42.1.c 22873013

[20] Sussman S Sun P Rohrbach LA Spruijt-Metz D One-year outcomes of a drug abuse prevention program for older teens and emerging adults: evaluating a motivational interviewing booster component. Health Psychol (2012) 31:476–85 10.1037/a0025756 21988096 PMC3276711

[21] Johnston LD O’Malley PM Bachman JG Schulenberg JE Monitoring the Future national survey results on drug use, 1975–2008: Volume I, Secondary school students (NIH Publication No. 09-7402). Bethesda, MD: National Institute on Drug Abuse (2009). p. 1–721

[22] Needle R Mccubbin H Lorence J Hochhauser M Reliability and validity of adolescent self-reported drug use in a family-based study: a methodological report. Int J Addict (1983) 18:901–12 6605944 10.3109/10826088309033058

[23] Graham J Flay B Johnson C Hansen W Grossman L Sobel J Reliability of self-report measures of drug use in prevention research: evaluation of the project smart questionnaire via the test-retest reliability matrix. J Drug Educ (1984) 14:175–93 10.2190/CYV0-7DPB-DJFA-EJ5U 6536737

[24] Stacy AW Flay BR Sussman S Brown KS Santi S Best JA Validity of alternative self-report indices of smoking among adolescents. Psychol Assess (1990) 2:442–6 10.1037/1040-3590.2.4.442

[25] Bloom BL A factor analysis of self-report measures of family functioning. Fam Process (1985) 24:225–39 10.1111/j.1545-5300.1985.00225.x 4018243

[26] Sussman S Dent CW One-year prospective prediction of drug use from stress-related variables. Subst Use Misuse (2000) 35:717–35 10.3109/10826080009148418 10807153

[27] Marin G Sabogal F Marin BV Otero-Sabogal R Perez-Stable EJ Development of a short acculturation scale for hispanics. Hisp J Behav Sci (1987) 9:183–205 10.1177/07399863870092005

[28] McCuller WJ Sussman S Dent CW Teran L Concurrent prediction of drug use among high-risk youth. Addict Behav (2001) 26:137–42 10.1016/S0306-4603(00)00082-4 11196288

[29] Sussman S Dent CW Stacy AW The relation of pro-drug use myths with self-reported drug use among youth at continuation high schools. J Appl Soc Psychol (1996) 26:214–67 10.1111/j.1559-1816.1996.tb01785.x

[30] Lau RR Hartman KA Ware JE Health as a value: methodological and theoretical considerations. Health Psychol (1986) 5:25–43 10.1037/0278-6133.5.1.25 3720718

[31] Reifman A Arnett JJ Colwell MJ Emerging adulthood: theory, assessment, and application. J Youth Dev (2007) 2:39–50

[32] Sussman S Mcculler WJ Dent CW The associations of social self-control, personality disorders, and demographics with drug use among high-risk youth. Addict Behav (2003) 28:1159–66 10.1016/S0306-4603(02)00222-8 12834658

[33] Radloff LS The CES-D scale: a self-report depression scale for research in the general population. Appl Psychol Meas (1977) 1:385–401 10.1177/014662167700100306

[34] Gambrill ED Richey CA An assertion inventory for use in assessment and research. Behav Ther (1975) 6:550–61 10.1016/S0005-7894(75)80013-X

[35] Wills TA Mcnamara G Vaccaro D Hirky AE Escalated substance use. J Abnorm Psychol (1996) 105:166–80 10.1037/0021-843X.105.2.166 8722998

[36] Tuinstra J Van Sonderen FLP Groothoff JW Van Den Heuvel WJA Post D Reliability, validity and structure of the Adolescent Decision Making Questionnaire among adolescents in The Netherlands. Pers Individ Dif (2000) 28:273–85 10.1016/S0191-8869(99)00096-3

[37] Victora CG Huttly SR Fuchs SC Olinto MT The role of conceptual frameworks in epidemiological analysis: a hierarchical approach. Int J Epidemiol (1997) 26:224–7 10.1093/ije/26.1.224 9126524

[38] Murray DM Hannan PJ Planning for the appropriate analysis in school-based drug-use prevention studies. J Consult Clin Psychol (1990) 58:458–68 2212183 10.1037//0022-006x.58.4.458

[39] SAS Institute Inc SAS/C Online Doc TM. Release 9.0. Cary, NC: SAS Institute (2000).

[40] Lemeshow S Hosmer DW Jr. A review of goodness of fit statistics for use in the development of logistic regression models. Am J Epidemiol (1982) 115(1):92–106 7055134 10.1093/oxfordjournals.aje.a113284

[41] Pampel FC Logistic Regression: A Primer. Sage University Papers Series on Quantitative Applications in the Social Sciences, 07-132. Thousand Oaks, CA: Sage Publications (2000).

[42] Sussman S Earleywine M Wills T Cody C Biglan T Dent CW The motivation, skills, and decision-making model of drug abuse prevention. Subst Use Misuse (2004) 39:1971–2016 10.1081/JA-200034769 15587955

[43] Sussman S Dent CW Stacy AW Craig S One-year outcomes of project towards no drug abuse. Prev Med (1998) 27:632–42 10.1006/pmed.1998.0420 9672959

[44] Sussman S Sun P Mcculler WJ Dent CW Project Towards No Drug Abuse: two-year outcomes of a trial that compares health educator delivery to self-instruction. Prev Med (2003) 37:155–62 10.1016/S0091-7435(03)00108-7 12855215

[45] Sampl S Kadden R Motivational Enhancement Therapy and Cognitive Behavioral Therapy for Adolescent Cannabis Users: 5 Sessions. Rockville, MD: Center for Substance Abuse Treatment, Substance Abuse and Mental Health Services Administration (2001).

[46] Walker DD Stephens R Roffman R Demarce J Lozano B Towe S Randomized controlled trial of motivational enhancement therapy with nontreatment-seeking adolescent cannabis users: a further test of the teen marijuana check-up. Psychol Addict Behav (2011) 25:474–84 10.1037/a0024076 21688877 PMC3177997

[47] Sussman S Dent CW Simon TR Stacy AW Galaif ER Moss MA Immediate impact of social influence-oriented substance abuse prevention curricula in traditional and continuation high schools. Drugs Soc (1995) 8:65–81 10.1300/J023v08n03_06

[48] Wills TA Cleary SD The validity of self-reports of smoking: analyses by race/ethnicity in a school sample of urban adolescents. Am J Public Health (1997) 87:56–61 10.2105/AJPH.87.1.56 9065227 PMC1380765

[49] Winters KC Stinchfield R Henly GA Schwartz RH Validity of adolescent self-report of alcohol and other drug involvement. Subst Use Misuse (1990) 25:1375–95 10.3109/10826089009068469 2132719

[50] Sussman S Dent CW Lichtman KL Project EX: outcomes of a teen smoking cessation program. Addict Behav (2001) 26:425–38 10.1016/S0306-4603(00)00135-0 11436934

[51] Grunbaum JA Lowry R Kann L Prevalence of health-related behaviors among alternative high school students as compared with students attending regular high schools. J Adolesc Health (2001) 29:337–43 10.1016/S1054-139X(01)00304-4 11691595

